# Supplementary material for: Reconstructing commuters network using machine learning and urban indicators
Source: Sci Rep. 2019 Aug 13;9:11801. doi: 10.1038/s41598-019-48295-x (PMC6692407; doi:10.1038/s41598-019-48295-x)
Supplement: Supplementary file 1 — Supplementary Information [file 41598_2019_48295_MOESM1_ESM.pdf]

Supplementary Information

Reconstructing commuters network using machine learning and urban indicators

Gabriel Spadon, Andre C. P. L. F. de Carvalho,
Jose F. Rodrigues-Jr, and Luiz G. A.Alves

List of Classifiers

#	Acronym	Name	Result
1	AdaBoost	Adaptive Boosting Classifier	✓
2	Bagging	Bagging Classifier	✓
3	BernoulliNB	Bernoulli Naive Bayes Classifier	✓
4	CalibratedCV	Calibrated Classifier with in-built cross-validation	✓
5	CatBoost	CatBoost Classifier	✓
6	ComplementNB	Complement Naive Bayes Classifier	✓
7	DecisionTree	Decision Tree Classifier	✓
8	ExtraTrees	Extremely Randomized Trees Classifier	✓
9	GaussianNB	Gaussian Naive Bayes Classifier	✓
10	GaussianProcess	Gaussian Processes Classifier	✗
11	GradientBoosting	Gradient Boosting Classifier	✓
12	HistGradientBoosting	Histogram-based Gradient Boosting Classification Tree	✓
13	KNeighbors	K-Nearest Neighbors Classifier	✓
14	LGBM	Light Gradient Boosting Machine Classifier	✓
15	LabelPropagation	Label Propagation Classifier	✗
16	LabelSpreading	Label Spreading Classifier	✗
17	LinearDA	Linear Discriminant Analysis Classifier	✓
18	LinearSVC	Linear Support Vector Classification	✓
19	Logistic	Logistic Regression Classifier	✓
20	LogisticCV	Logistic Regression Classifier with in-built cross-validation	✓
21	MLP	Multi-layer Perceptron Classifier	✓
22	MultinomialNB	Multinomial Naive Bayes Classifier	✓
23	NearestCentroid	Nearest Centroid Classifier	✓
24	NuSVC	Nu-Support Vector Classification	✗
25	PassiveAggressive	Passive Aggressive Classifier	✓
26	Perceptron	Perceptron Classifier	✓
27	QuadraticDA	Quadratic Discriminant Analysis Classifier	✓
28	RadiusNeighbors	Radius Neighbors Classifier	✗
29	RandomForest	Random Forest Classifier	✓

30	Ridge	Ridge Classifier	✓
31	RidgeCV	Ridge Classifier with in-built cross-validation	✓
32	SGD	Stochastic Gradient Descent Classifier	✗
33	SVC	Support Vector Classification	✗
34	XGBoost	Extreme Gradient Boosting Classifier	✓
Number of selected classifiers:			27/34

Table 1: List of classifiers tested during the unweighted link prediction of the commuters network. The *Acronym* column presents the short name of the algorithms, the *Name* column shows the full name of the algorithms, and the *Results* column marks with ✓ the classifiers that do not require early hyperparameter tuning and have passed for the subsequent testing phase, and with ✗ the discarded ones.

List of Regressors

#	Acronym	Name	Result
1	ARD	Automatic Relevance Determination Regression	✗
2	AdaBoost	Adaptive Boosting Regressor	✓
3	Bagging	Bagging Regressor	✓
4	BayesianRidge	Bayesian Ridge Regressor	✓
5	CCA	Canonical Correlation Analysis Regressor	✗
6	CatBoost	CatBoost Regressor	✓
7	DecisionTree	Decision Tree Regressor	✓
8	ElasticNet	Elastic-Net Regressor	✓
9	ElasticNetCV	Elastic-Net Regressor with in-built cross-validation	✓
10	ExtraTrees	Extremely Randomized Trees Regressor	✓
11	GaussianProcess	Gaussian Processes Regressor	✗
12	GradientBoosting	Gradient Boosting Regressor	✓
13	HistGradientBoosting	Histogram-based Gradient Boosting Regression Tree	✓
14	Huber	Huber Regressor	✓
15	Isotonic	Isotonic Regression	✗
16	KNeighbors	K-Nearest Neighbors Regressor	✓
17	KernelRidge	Kernel Ridge Regressor	✗
18	LGBM	Light Gradient Boosting Machine Regressor	✓
19	Lars	Lars Regressor	✗
20	LarsCV	Lars Regressor with in-built cross-validation	✗
21	Lasso	Lasso Regressor	✗
22	LassoCV	Lasso Regressor with in-built cross-validation	✓
23	LassoLars	Lasso-Lars Regressor	✗
24	LassoLarsCV	Lasso-Lars Regressor with in-built cross-validation	✓
25	LassoLarsIC	Lasso-Lars Regressor with information criterion	✓
26	Linear	Linear Regression	✗
27	LinearSVR	Linear Support Vector Regression	✗
28	MLP	Multi-layer Perceptron Regressor	✓
29	NuSVR	Nu-Support Vector Regression	✗

30	OrthogonalMP	Orthogonal Matching Pursuit Regrssor	✓
31	OrthogonalMPCV	Orthogonal Matching Pursuit Regrssor with in-built cross-validation	✗
32	PLSCanonical	Partial Least Squares Canonical Regressor	✗
33	PLS	Partial Least Squares Regressor	✓
34	PassiveAggressive	Passive Aggressive Regressor	✗
35	RANSAC	Random Sample Consensus Regressor	✗
36	RadiusNeighbors	Radius Neighbors Regressor	✗
37	RandomForest	Random Forest Regressor	✓
38	Ridge	Ridge Regressor	✓
39	RidgeCV	Ridge Regressor with in-built cross-validation	✓
40	SGD	Stochastic Gradient Descent Regressor	✗
41	SVR	Support Vector Regression	✗
42	TheilSen	Theil-Sen Regressor	✗
43	TransformedTarget	Transformed Target Regressor	✗
44	XGBoost	Extreme Gradient Boosting Regressor	✓
Number of selected regressors:			23/44

Table 2: List of regressors tested during the weighted link prediction of the commuters network. The *Acronym* column presents the short name of each algorithm, the *Name* column shows the full name of all algorithms, and the *Results* column marks with ✓ the regressors that do not require early hyperparameter tuning and have passed for the subsequent testing phase, and with ✗ the discarded ones.

Classifiers' Performance

#	Algorithm	Mean	Standard Deviation (σ)	Variance (σ^2)
1	CatBoost	0.87938	0.00270	0.00001
2	XGBoost	0.87523	0.00282	0.00001
3	LGBM	0.87391	0.00306	0.00001
4	HistGradientBoosting	0.87382	0.00298	0.00001
5	GradientBoosting	0.87288	0.00298	0.00001
6	AdaBoost	0.85866	0.00468	0.00002
7	Bagging	0.85620	0.00523	0.00003
8	RandomForest	0.83895	0.01334	0.00018
9	DecisionTree	0.81229	0.00924	0.00009
10	ExtraTrees	0.74033	0.02400	0.00058
11	LogisticCV	0.70626	0.02802	0.00079
12	Ridge	0.70278	0.01123	0.00013
13	LinearDA	0.69882	0.01014	0.00010
14	Logistic	0.67249	0.05846	0.00342
15	MLP	0.64219	0.02921	0.00085
16	QuadraticDA	0.63175	0.00419	0.00002
17	BernoulliNB	0.62663	0.00316	0.00001
18	KNeighbors	0.61093	0.00778	0.00006
19	LinearSVC	0.59470	0.06886	0.00474

20	CalibratedCV	0.58282	0.04873	0.00237
21	NearestCentroid	0.57061	0.00853	0.00007
22	GaussianNB	0.56790	0.01159	0.00013
23	ComplementNB	0.56444	0.04092	0.00167
24	MultinomialNB	0.56191	0.04357	0.00190
25	PassiveAggressive	0.52767	0.03754	0.00141
26	RidgeCV	0.51654	0.07597	0.00577
27	Perceptron	0.50213	0.02163	0.00047

Table 3: Statistics from the bootstrap sampling describing the *Mean*, *Standard Deviation*, and *Variance* of a thousand predictions made on different random samples of the training set. The experiment was carried out using the classifiers that have passed the first testing phase, see Table 1.

Regressors' Performance

#	Algorithm	Mean	Standard Deviation (σ)	Variance (σ^2)
1	XGBoost	0.65623	0.01124	0.00013
2	GradientBoosting	0.65430	0.01139	0.00013
3	LGBM	0.63696	0.01102	0.00012
4	HistGradientBoosting	0.63630	0.01102	0.00012
5	ExtraTrees	0.62010	0.01405	0.00002
6	RandomForest	0.60985	0.01727	0.00003
7	Bagging	0.60943	0.01675	0.00028
8	CatBoost	0.58423	0.01269	0.00016
9	AdaBoost	0.49586	0.04169	0.00174
10	MLP	0.49051	0.04793	0.00230
11	BayesianRidge	0.47401	0.00049	0.00002
12	ElasticNetCV	0.47311	0.00047	0.00002
13	LassoLarsCV	0.47300	0.00571	0.00003
14	LassoCV	0.47268	0.00529	0.00003
15	RidgeCV	0.47175	0.00604	0.00004
16	LassoLarsIC	0.47120	0.00629	0.00004
17	OrthogonalMP	0.47020	0.00511	0.00003
18	Ridge	0.46906	0.00639	0.00004
19	Huber	0.42919	0.01558	0.00024
20	PLS	0.36433	0.03680	0.00135
21	KNeighbors	0.34232	0.01488	0.00022
22	DecisionTree	0.31286	0.05175	0.00268
23	ElasticNet	0.20615	0.03905	0.00153

Table 4: Bootstrap sampling performance describing the *Mean*, *Standard Deviation*, and *Variance* of a thousand predictions made on different random samples of the training set. The experiment was carried out using the regressors that have passed the first testing phase, as described in Table 2.

List of Features and Selected Features

		Classifier		Regressor	
#	Features	City_s	City_t	City_s	City_t
1	Area	✓	✓	✓	✓
2	Child labor	✓	✗	✗	✓
3	Domestic violence	✗	✗	✗	✗
4	Elderly population	✗	✓	✓	✓
5	Female population	✓	✗	✓	✗
6	Gross Domestic Product	✗	✓	✗	✓
7	Homicides	✗	✗	✓	✗
8	Human Development Index	✓	✓	✗	✓
9	Illiteracy	✓	✓	✓	✗
10	Income	✗	✓	✗	✓
11	Male population	✗	✗	✗	✗
12	Minimum wage	✗	✓	✓	✓
13	Population	✗	✗	✗	✗
14	Population density	✓	✗	✓	✓
15	Sanitary sewage	✗	✓	✗	✓
16	Sanitation	✗	✗	✗	✗
17	Street arborization	✓	✓	✓	✓
18	Street urbanization	✓	✗	✓	✗
19	Suicides	✗	✗	✗	✓
20	Traffic accidents	✗	✓	✗	✓
21	Tuition rate	✓	✗	✗	✗
22	Unemployment	✓	✗	✓	✗
23	Distance	✓		✓	
Number of selected features:		21/45		23/45	

Table 5: List of features selected by the threshold-based feature selection process applied to both models inferred from the XGBoost classifier and regressor. In the table, we use City_s and City_t to denote the source and target cities, ✓ to indicate the remaining features after feature selection and ✗ to indicate the removed ones. Notice that the distance between two given cities is the same regardless of the city of departure. As a consequence, the distance represents a single feature, and there is no distinction between the distance from the source to the target city and vice-versa.
